# Resveratrol-loaded invasome gel: A promising nanoformulation for treatment of skin cancer

**DOI:** 10.1007/s13346-024-01534-9

**Published:** 2024-02-15

**Authors:** Bassant Samir, Amal El-Kamel, Noha Zahran, Lamia Heikal

**Affiliations:** 1 Department of Pharmaceutics, Faculty of Pharmacy, Alexandria University, 1 Khartoum Square, Azarita, P.O. Box 21521, Alexandria, Egypt; 2 Department of Histology and Cell Biology, Faculty of Medicine, Alexandria University, Alexandria, Egypt

**Keywords:** Terpenes, Skin permeation, Cytotoxicity, Cellular uptake, Anticancer activity, Caspase-3

## Abstract

Skin cancer is a widespread type of cancer representing 30% of all cancer types worldwide. Resveratrol (RSV) is an anticancer drug used for skin cancer treatment. Several limitations of RSV such as poor aqueous solubility, first-pass metabolism, and instability limit their topical use. The study aimed to develop and optimize RSV-loaded invasomes for topical administration as well as assess their efficacy in vivo. The optimized RSV-loaded invasomes showed small particle size (208.7 ± 74 nm), PDI (0.3 ± 0.03), high % entrapment efficiency (77.7 ± 6%), and negative zeta potential (−70.4 ± 10.9 mV). They showed an initial burst effect followed by controlled drug release for 24 h. RSV-loaded invasomal gel revealed the highest skin deposition percentage (65%) in ex vivo rat skin, the highest potency (low IC_50_ of 6.34 μg/mL), and the highest cellular uptake when tested on squamous cancerous cells (SCCs) when compared to other formulations. The antitumor effect of topical RSV-loaded invasomes was also evaluated in vivo in Ehrlich-induced mice models. The results revealed that RSV-loaded invasomal gel exhibited the smallest tumor volume with no signs of organ toxicity indicating its safety in skin cancer treatment. Upregulation of BAX and Caspase-3 gene levels and downregulation of NF-kB and BCL2 protein levels were demonstrated using RT-PCR and ELISA tests, respectively. Interestingly, the present study is the first to develop RSV-loaded invasomal gel for topical skin cancer treatment. According to our results, invasomes are considered promising lipid-based nanosystems for topical RSV delivery having high skin penetration ability and anticancer effect in the treatment of skin carcinoma.

**Graphical Abstract:**

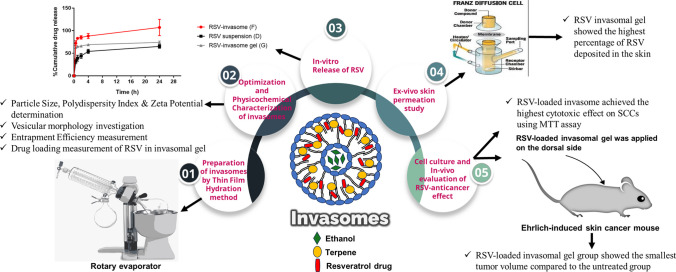

## Introduction

Skin cancer is one of the challenging malignancies that affect people’s lives, representing 30% of all cancer types all over the world. Melanoma and non-melanoma including its 2 subtypes: basal cell carcinoma (BCC) and squamous cell carcinoma (SCC) are the most significant types of skin cancer (NMSC) [1, 2].

Several factors are responsible for the development of skin cancer such as chronic UV exposure, history of skin cancer, or sunburns. UV radiation being the most important cause of skin cancers plays a crucial role in the initiation, promotion, and progression phases of the carcinogenesis process [2, 3]. Chronic exposure to UV causes release of inflammatory mediators like TNF-α and high reactive oxygen species (ROS) levels which interact with DNA, and proteins causing cellular damage and angiogenesis in the skin that result in the growth of tumors [3, 4].

Concerning the treatment of skin cancer, the goal of anticancer therapy is to reduce dermatological carcinogenic lesions via induction of apoptosis, suppression of cancer cell growth, and inhibition of angiogenesis [5]. Despite the presence of different treatment therapies to treat skin cancer, the most important challenge facing such therapies is the absence of an efficient treatment plan and the absence of a proficient drug delivery system as drug localization in the skin is of critical importance in the treatment of skin cancer.

Topical delivery has several advantages over different routes of administration, especially the oral route. It provides sustained drug delivery and bypasses liver metabolism, thus reducing the side effects of drugs [6]. However, inhibition of drug permeation through the skin via the stratum corneum (SC), the outermost layer of the skin, represents a major challenge facing topical drug delivery [7].

One of the current approaches is to overcome the barrier function of SC by using various delivery systems for topical drugs including nanodrug delivery systems such as nanoemulsions, liposomes, solid lipid nanoparticles, and nanostructured lipid carriers [8]. Novel nanodrug delivery systems have been adopted recently as promising delivery systems to enhance drug penetration through the skin such as niosome (prepared mostly by non-ionic surfactant and cholesterol) and ethosome (containing high amounts of ethanol in their structure) [9].

Compared to these systems, invasomes are flexible deformable liposomal vesicular systems containing a mixture of nontoxic natural components: phospholipids (PC), a terpene or a mixture of terpenes, and ethanol with improved skin permeation in comparison with liposomes [10]. The synergistic effect between terpenes, ethanol, and phospholipids (PC) provides exceptional flexibility to the vesicles with improved permeation across the stratum corneum as both ethanol and terpenes act as permeation enhancers. Thus, the rate of penetration of invasomes through the skin is significantly greater than that of liposomes and ethosomes. This is considered a very useful approach in improving the transdermal delivery of drugs especially anticancer drugs for the management of skin cancer [7, 11].

Although penetration enhancers work efficiently in transdermal delivery, few are clinically authorized owing to their skin irritation and toxicity. Since this is considered an important regulatory requirement upon developing a formulation for human use, the objective of our study was to balance the potency and toxicity of different ingredients used in the development of an invasome formulation.

Terpenes, a main component of invasomes, are generally recognized as safe (GRAS) with respect to the Food and Drug Administration [9]. They are potent penetration enhancers; however, the toxicity of terpenes is a major concern and should be considered [12]. Thymol is a natural terpene, one of the major constituents of essential oils of thyme. It is not a commonly used terpene in the preparation of invasomes compared to other terpenes such as cineole and limonene. Thymol has been used once by Kaltschmidt et al. [13] in invasomal preparation for its antibacterial effect. It has been used in our study for the first time owing to its safety and lack of toxicity as well as its anticancer effect [14].

Resveratrol (RSV) (3, 5, 4’-tri-hydroxystilbene) is a natural polyphenol drug found in grapes, red wine, and other fruits. It possesses several therapeutic effects such as antiinflammatory, antiaging, antioxidant, and chemotherapeutic properties [15]. RSV’s anticancer effect was approved and tested on skin cancer in mice [16, 17]. Its anticancer mechanism of action was studied in all cancer stages where it inhibits angiogenesis pathways, cancer growth, and inflammation and induces apoptosis in cancer cells via regulating signal transduction pathways [18]. As an anticancer agent, it inhibits the growth of squamous cell carcinoma, breast, liver, colon, and many other types of cancer [19]. However, RSV possesses low oral bioavailability due to hepatic metabolism, low water solubility, high lipophilicity, and poor stability in aqueous physiological solutions [20, 21]. It belongs to class II in the Biopharmaceutical Classification System (BCS) [22]. It is worth mentioning that dermal delivery of RSV has been studied widely using different nanoformulation approaches such as ethosomes, liposomes, and deformable liposomes [23–25].

To our knowledge, RSV was not loaded in an invasomal preparation before. Thus, the aim of this study was to formulate RSV, for the first time in an entirely safe naturally-based lipid vesicular system; RSV-loaded invasome containing thymol as terpene. In addition to optimizing its method of preparation and applying it in the form of a gel for the management of skin cancer. The molecular mechanism of the RSV-loaded invasomes in the treatment of skin cancer in vivo in Erlich-induced skin cancer mice model was also elucidated.

## Materials and methods

### Materials

Resveratrol (RSV) (high purity > 98%) was purchased from Guangzhou Phytochem Sciences Inc. (Guangzhou, China). Thymol was purchased from Oxford Lab Fine Chem I.I.P (India). Dialysis bags (molecular weight cut off 12,000–14,000 Da) (Serva, Heidelberg, Germany). Coumarin-6 dye was obtained from Sigma-Aldrich (St. Louis, MO, USA). 3-(4,5-dimethylthiazol-2-yl)-2,5-diphenyltetrazolium bromide (MTT) was supplied by SERVA Electrophoresis GmbH (Germany). TRIzol^®^ Reagent was provided by Invitrogen, Thermo Fisher Scientific (Waltham, MA, USA). The primers were acquired from Sigma-Aldrich (UK) and a one-step RT qPCR kit (SYBR Green with low ROX) was purchased from Intron Biotechnology, Korea. B-cell Lymphoma/Leukemia 2 ELISA kit was purchased from Novus Biologicals, UK (NBP2-69946), and the Nuclear Factor Kappa B ELISA kit was obtained from Wuhan Fine Biotech Co., Ltd (Wuhan, Hubei, China). Acetonitrile and DMSO (HPLC grade) were purchased from Fischer Scientific (Loughborough, UK). Methanol, chloroform, ethanol, and formalin were provided by Al-Gomhureya Co. (Cairo, Egypt). All other reagents and chemicals were of analytical grade.

### Preparation and optimization of RSV-loaded invasomes

#### Preparation of invasomes

Invasomes were prepared by thin film hydration method with some modifications [7]. Five milligrams (5 mg) of RSV, 100 mg soybean phosphatidylcholine (PC), and 100 mg thymol (terpene) were dissolved in a 10 mL mixture of chloroform and methanol (2:1) in a dry clean round bottom flask to achieve the final RSV concentration of 0.5 mg/mL. The organic solvent was then evaporated using a rotary evaporator (Buchi AG, Switzerland) under vacuum using an oil-free vacuum pump (ROCKER^®^, Model Rocker 801, Taiwan) for around 25 min at 60 °C (above transition temp. of lipid) (Tc) until a thin uniform film was formed. Ten milliliters of hydration solution (0.5: 9.5 v/v ethanolic PBS, pH 7.3) was added to the film and left on the rotary evaporator at room temperature for 30 min. Unloaded invasomes were prepared using the same method but without adding RSV.

#### Optimization of the prepared invasome formulation

To achieve an optimum formulation with reduced particle size and uniform vesicular structure, different methods of particle size reduction were assessed such as homogenization, probe sonication, and extrusion. During sample homogenization, a high shear homogenizer (Ultra Turrax; IKA Labortechnik, Staufen, Germany) was used at different speeds; 5000, 6000, 7000, and 10,000 rpm. Homogenization was carried out in an ice bath for 3 cycles, 1.5 min or 3 min each with a constant rest time of 5 min between each cycle. As for probe sonication, samples were subject to sonication (60% amplitude for 5 min with a pulse 2 s on, 2 s off). As for the extrusion method, the samples were subject to 5, 9, or 11 cycles. Regardless of the method used for particle size reduction, all samples were left overnight at 4 °C in the fridge to stabilize the vesicles until further characterization.

### Physicochemical characterization of invasomes

#### Particle size, polydispersity index, and zeta potential determination

Samples were first diluted with deionized water 1:50 dilution and sonicated (ELMA, Germany) for 5 min before being measured. Samples were measured using Malvern Zetasizer Nano-ZS, Malvern instrument (Malvern, UK) at 25 °C and 173° angle to assess particle size (PS), polydispersity index (PDI), and zeta potential (ZP). Measurements were done in triplicate and all results were expressed as mean value ± (SD) [26].

#### Transmission electron microscopy (TEM)

The vesicular morphology of RSV-loaded invasomes and unloaded invasomes was investigated using a transmission electron microscope (TEM) (JEM-1400 Plus 120 kV, Joel, Peabody, MA, USA) [26]. Both samples were first diluted with deionized water (1:50) and bath sonicated for 5 min at room temperature. Samples were then added to a carbon-coated grid and stained with 1% uranyl acetate for 30 s. Samples were left to air-dry and were then examined at 20 K × magnification power [27].

#### Percentage entrapment efficiency and drug loading measurement (EE% and DL%)

To measure the amount of RSV entrapped in the prepared invasomes, samples were centrifuged at 14,000 rpm for 1 h at 4 °C (Model3K-30; Sigma Laborzentrifugen GmbH, Osterode, Germany). The supernatant was then separated and used to quantitatively measure the amount of free unentrapped RSV using an ultraviolet and visible spectrophotometer at *λ*_max_ = 305 nm (Cary 60 UV-Vis Spectrophotometer, Agilent, Santa Clara, CA, USA). Measurements were done in triplicate and represented as mean value ± SD. EE% was calculated using the following Eq. (1) [27]:

1 $$\%\;EE\;=\;\frac{Total\;theoretical\;RSV\;content\;\left(mg\right)\;-\;unentrapped\;RSV\;content\;(mg) }{Total\;theoretical\;RSV\;content\;(mg)}\;\times\;100$$

Drug loading was calculated using the following Eq. (2):

2 $$\%\;DL\;=\;\frac{Actual\;drug\;content}{Total\;weight\;of\;invasomes}\;\times\;100$$

### Formulation of invasome-loaded gel

To increase contact time on the skin, the optimum prepared invasome formulation was loaded in a gel as developed by Teaima et al*.* [28] with slight modifications. The gel was prepared by dispersing Carbopol (Carbomer 941) polymer (3% w/v) in the invasomal dispersion (10 mL) and left on a magnetic stirrer (IKA Labortechnik, Staufen, Germany) at 25 °C and 800 rpm till complete dispersion. The dispersion was then neutralized by adding triethanolamine (TEA) dropwise until a soft viscous gel was obtained [28].

#### Characterization of invasomal gel

##### Drug content in the gel

To calculate drug loading of RSV in the gel, 1 gm of RSV-loaded invasomal gel was dissolved in 20 g ethanol by heating on a magnetic stirrer (IKA Labortechnik, Staufen, Germany) at 70 °C and 1200 rpm for 30 min [29]. To break down the vesicles, the sample was subjected to probe sonication (Bandelin, Berlin, Germany) (60% amplitude continuously for 15 min) and then centrifuged at 14,000 rpm at 21 °C for 10 min to get the supernatant where drug content was analyzed by UV-Vis spectrophotometry at *λ*_max_ = 305 nm.

##### Spreadability and rheological measurement

The spreadability of loaded and unloaded gels was measured by spreading 0.5 g gel on a 2 cm diameter circle pre-marked on a flat glass plate after which a second glass plate was employed. A weight of 500 gm was allowed to rest on the upper glass plate for 5 min after which the diameter of the circle formed after spreading of the gel was measured [30].

As for rheological measurement, the shear thinning and thixotropic ability of both loaded and unloaded gels was performed by measuring the viscosity with a Viscometer (Brookfield RV head multipoint viscometer with spindle CP-52). A sufficient amount of gel was added to the receiving chamber at room temperature where the gel was rotated at 0.1, 0.5, 1, 5, 1, 0.5, and 0.1 rotations per minute.

### In vitro release of RSV

In vitro drug release was carried out using the dialysis bag method [27]. Constant sample volumes of 1 mL RSV suspension in PBS (D), 1 mL optimized RSV-loaded invasome liquid formulations (F2), and 1 g RSV-loaded invasomal gels (G) were added into dialysis bags (molecular weight cut off 12,000–14,000 Da) (Serva, Heidelberg, Germany) with an amount equivalent to 0.5 mg/mL drug. The bags were tightly sealed from both sides and immersed in 30 mL phosphate buffer saline (pH 7.3) as a release medium to ensure sink conditions (solubility of RSV in PBS pH 7.3 was 100 µg/mL). All samples were placed in a shaking water bath maintained at 37 ± 0.5 °C and 100 rpm. One milliliter of samples was withdrawn at different sampling time intervals (0.5, 1, 2, 4, 6, and 24 h) and replaced by equal volumes (1 mL) of fresh PBS to maintain sink conditions. Withdrawn samples were then analyzed spectrophotometrically at *λ*_max_ = 305 nm. Percent cumulative drug release (mean ± SD) was plotted against time after correction where % cumulative drug released was calculated using the following Eqs. (3) and (4) [31]:

3 $$Amount\;of\;drug\;release\;\left(mg/mL\right)\;=\;\frac{Conc.\;\times\;Volume\;of\;release\;medium\;\times\;DF}{100}$$

4 $$\%\;Cumulative\;drug\;release\;=\;\frac{Sample\;volume\;taken\;(ml)}{Bath\;volume\;(v)}\;\times\;P\;\left(t\;-\;1\right)\;+\;Pt$$

In this equation: P(t-1) is the percentage of the amount released before time (t) and Pt is the percentage of the amount released at time (t).

### Ex vivo drug deposition

The ex vivo drug permeation/deposition study on animal skin was approved by the Ethics Committee of Medical Research, Faculty of Pharmacy, Alexandria University, according to the requirements of the Institutional Animal Care and Use Committee (IACUC), Alexandria University, Egypt (AU-06-2022-1191133). This study was applied on rat skin using Franz diffusion cells with some modifications [7]. The full-thickness dorsal skin was isolated from Wistar rats (average 200 g weight), dorsal hair was shaved, the subcutaneous tissue was removed, and the skin was washed in saline. The skin was mounted on Franz cells between donor and receiver cells with the epidermal layer facing the donor cell and the dermis inner layer facing the receptor cell. The receiver compartment was filled with 20 mL ethanolic PBS (3:7) to ensure sink conditions. The donor cells were filled with 1 mL drug suspension in PBS (D), 1 mL optimized RSV-loaded invasomes dispersion (F2), or 1 g RSV-loaded invasomal gels (G) where the final drug concentration in each sample was equivalent to 0.5 mg/mL over a skin diffusion area of 1 cm^2^. Samples were placed in a shaking water bath at 32 ± 0.5 °C and 80 rpm. Samples (0.5 mL) were then withdrawn from receptor cells at different time intervals (0.5, 1, 2, 4, and 6 h) and replaced with 0.5 mL fresh ethanolic PBS (3:7) to maintain constant volume and sink condition. The amount of RSV was analyzed by reversed phase isocratic HPLC (Agilent Technologies-1260 Infinity, Germany) with a UV detector (G1314F, C18 column (Agilent HC-C18 [4.6 × 250 mm], and 5 μm particle size)) and Agilent ChemStation^®^ software (32-bit version) (revision B.02.01 SR1). The mobile phase used was acetonitrile-deionized water mixture (40:60 v/v) at a 1.0 mL/min flow rate at room temperature. An aliquot of 100 μL sample was injected and detected at (*λ*_max_) 305 nm [32].

At the end of the study, the amount of RSV deposited in the skin was measured where skin samples were collected at the end of the experiment, gently rinsed with the release medium, and immersed in a methanol/PBS mixture 1:1 then thoroughly homogenized (Ultra Turrax; IKA Labortechnik, Germany) for complete drug extraction. Digested samples were then centrifuged, and the supernatants were filtered via a 0.22 um syringe filter before HPLC quantification.

The amount of drug permeated through the skin and deposited in the skin layers was carried out in triplicate and the results were presented as means ± SD.

### Ex vivo cell culture

#### Cell culture

All cell culture experiments were carried out at the Centre of Excellence for Research in Regenerative Medicine and its Applications (CERRMA), Faculty of Medicine, Alexandria University, Alexandria, Egypt. Squamous skin carcinoma cells (SCCs) of passage number 25 were grown in DMEM high glucose cell culture media supplemented with 100 U/mL penicillin-streptomycin and 10% (v/v) FBS in a humidified environment of 5% CO_2_.

#### Cytotoxicity test

Cytotoxicity of RSV-loaded invasome, unloaded invasome, and RSV solution in ethanol was assessed using 3-(4,5-dimethylthiazol-2-yl)-2,5-diphenyltetrazolium bromide (MTT) assay where IC_50_ of RSV was calculated in each of the prepared formulae [27]. Briefly, SCCs were cultured in 96-well plates (Greiner Bio-One, Germany) at a seeding density of 5 × 10^3^ cells/well and incubated at 37 °C for 24 h to ensure cell adherence to the plates. The cells received serial dilutions of RSV-loaded, RSV-unloaded invasomes or RSV solution in ethanol (concentration range 0–50 ug/mL) and left to incubate for 48 h. The medium was then removed and replaced with MTT solution in PBS (5 mg/mL). The cells were further incubated for 4 h in the dark at 37 °C and 5% CO_2_. MTT solution was then replaced with 100 µL of DMSO and left for 10 min with gentle shaking on an orbital shaker (Heidolph Instruments, Schwabach, Germany) to dissolve the formed purple formazan crystals. Absorbance was measured using an automated microplate reader (BioTekR Instruments, VT, USA) at 570 nm to calculate the percentage of cell viability with respect to control untreated cells, in triplicate using Eq. (5) [27]. Half of the maximum inhibitory concentration (IC_50_) of RSV was calculated using a dose-response curve with non-linear regression analysis using GraphPad Prism (version 9).

5 $$\%\;Cell\;viability\;=\;\frac{Mean\;absorbance\;of\;RSV-treated\;cells}{Mean\;absorbance\;of\;RSV-untreated\;cells}\;\times\;100$$

#### Cellular uptake

Cellular uptake was assessed using invasomes loaded with coumarin-6 fluorescent instead of RSV prepared using thin film hydration method (0.5 mg/mL) and free coumarin-6 solution (C6) in ethanol (0.5 mg/mL). SCCs were cultured at 5 × 10^5^ cells/well in a 6-well plate and then incubated for 24 h. After incubation, coumarin-6-loaded invasome and free coumarin-6 solution were applied to the cells to reach a final concentration of 100 ng/mL and incubated for a further 4 h. Cells were washed 3 times with PBS then fixed with 4% v/v paraformaldehyde in PBS solution at room temperature and left for 15 min. Cellular uptake was assessed by measuring the fluorescence intensity of C6 fluorescent dye using a confocal laser scanning microscope (CLSM) at 355 nm (LeicaR Microsystems Inc. Model DMi8, Metzler, Germany). All experiments took place away from direct light to prevent the detrimental effects of ambient light. The fluorescence intensity in images produced by CLSM was quantified using ImageJ 1.52a software acquired by the National Institutes of Health, USA to determine fluorescence intensity [27].

### In vivo evaluation of anticancer efficacy of the prepared formulae

#### Animals

Forty male Swiss Albino CD1 mice weighing 18–25 gm each were purchased, housed, and maintained at the animal house of Medical Research Institute, Alexandria University, Egypt. Mice were kept under ambient conditions at 25 ± 1 °C and 50% relative humidity with dark/night cycle for 12 h. They had free access to food and water throughout the study. This in vivo study on animals was approved by the Ethics Committee of Medical Research, Faculty of Pharmacy, Alexandria University, according to the requirements of the Institutional Animal Care and Use Committee (IACUC), Alexandria University, Egypt (approval number) (AU-06-2022-1191133). An Ehrlich ascites cancerous (EAC) cells mouse was obtained from the National Institute of Cancer, Egypt. The mice’s back skin was shaved before tumor induction and treatment.

#### Tumor induction

Ehrlich ascites–induced skin cancer mice model was used to induce skin cancer in Swiss Albino male CD1 mice. The Ehrlich ascites cancerous cells (EAC) were obtained from the ascitic fluid–bearing tumor of a mouse (Swiss albino) which was cultured inside the mouse for 8–10 days. Skin cancer was induced by intradermal injection of 100 µL Ehrlich ascites cancerous cells (EAC) (containing approximately 1*10^6^ of tumor cells) in the dorsal side [33] with some modifications. Forty mice were divided into 5 groups, 8 mice in each group (*n* = 8), as follows: group 1 (Gp1) for healthy negative control mice, group 2 (Gp2) for positive control (untreated cancerous) mice, group 3 (Gp3) for blank gel-treated mice, group 4 (Gp4) for unloaded invasomal gel-treated mice, and group 5 (Gp5) for drug-loaded invasomal gel-treated mice.

Treatment started 10 days after tumor induction where 0.5 g gel was applied topically on mice skin daily (equivalent to 1 mg/kg RSV) over a 1 cm diameter area on the dorsal side of each mouse and covered with bandage and plaster to decrease leakage of gel. The treatment duration post-induction was 21 days. During the treatment period, mice weights and tumor sizes were measured every other day using a balance and a caliber respectively.

#### Skin tumor volume measurement

Skin tumor size was measured for all mice groups except gp 1 (healthy) every other day using a caliper tool where length and width were determined to evaluate the antitumor effect of the treatment formulae compared to the untreated positive group (gp 2). Length and width measurements of tumors were presented as mean ± SD. Mean tumor volume was calculated as shown in the following Eq. (6):

6 $${Mean\;tumor\;volume\;=\;0.5X}^{2}Y$$

where *X* and *Y* are the width and length diameters, respectively. Percent change in tumor volume is represented in the following Eq. (7) [34]:

7 $$\%\;change\;in\;tumor\;volume\;=\;\frac{mean\;tumor\;volume\;of\;positive\;control\;group-mean\;tumor\;volume\;of\;treated\;group }{Mean\;tumor\;volume\;of\;positive\;control\;group}\;\times\;100$$

#### Termination

At the end of the treatment, mice were terminated by exsanguination after exposure to a high dose of inhaled isoflurane. Blood was collected from the orbital sinus of the eye and the collected blood samples were centrifuged at 3000 rpm at 4 °C for 10 min to separate plasma. Plasma was then stored at −80 °C till further biochemical analysis. The tumor was collected where parts were snap-frozen, while others were put in 10% v/v formalin for histological examination. The frozen parts were maintained at −80 °C for further RT-PCR and ELISA examination. Other organs such as the kidney, liver, and lungs were isolated and kept in 10%v/v formalin for toxicity assessment.

#### Quantitative analysis of cancer biomarkers on gene level using PCR and protein level using ELISA

PCR experiment was carried out in the Pharmaceutics Molecular Laboratory, Department of Pharmaceutics, Faculty of Pharmacy, Alexandria University.

Expression levels of BAX and Caspase-3 genes were quantified using polymerase chain reaction (PCR) to evaluate the effect of different formulations (blank gel, unloaded invasomal gel, and RSV-loaded invasomal gel) on the treatment of skin cancer in comparison to negative and positive control groups [35]. First, snap-frozen samples were homogenized with TRIZOL reagent (Invitrogen, USA). An easy spin RNA extraction kit (Intron Biotechnology, India) was used in the purification and extraction of RNA [27]. A NanoDrop ND-1000 (NanoDrop DS-11 FX; DeNovix, Delaware, USA) was used to measure the quality and concentrations of RNA at 260 nm and 280 nm absorbance. Primers used for BAX [35], Caspase-3 [27], and GAPDH are represented in Table 1. GAPDH was used as a housekeeping gene to normalize relative transcript levels. According to the guidelines of Applied Biosystems/Life Technologies, the method applied in PCR quantification for BAX and Caspase-3 expressions was the comparative threshold cycle (2^−ΔΔCt^) method [27]. Results were normalized to GAPDH expression and expressed as arbitrary units.

**Table 1:** Forward and reverse primers of BAX and Caspase-3 genes for RT-PCR test for in vivo experiment

Gene	Forward	Reverse
BAX	GCTGACATGTTTGCTGATGG	GATCAGCTCGGGCACTTTAG
Caspase-3	AGGGGTCATTTATGGGACA	TACACGGGATCTGTTTCTTTG
GAPDH	TCACCACCATGGAGAAGGC	GCTAAGCAGTTGGTGGTGCA

Protein levels of B-cell lymphoma/leukemia 2 (Bcl2) and nuclear factor kappa B (NF-kB) were measured in isolated tumors using enzyme-linked immunosorbent assay (ELISA) using B-cell Lymphoma/Leukemia 2 ELISA kit and Nuclear Factor Kappa B ELISA kit, respectively. Frozen samples were homogenized in PBS to reach a homogenate concentration of 10% w/v. ELISA kits were used for measurements according to the manufacturer’s protocol.

### Histological examination

Tumors preserved in formalin (10% v/v) were integrated into paraffin wax blocks. Hematoxylin and eosin (H&E) stain was used to stain the samples to be examined histologically under the light microscope (Carl Zeiss, Köln, Germany) accompanied by a Canon digital camera. Histological samples were done to evaluate the antitumor effect of the treated formulations compared to the untreated group.

### Blood biochemical assay to assess toxicity

Separated plasma was used to quantify levels of urea, creatinine, alanine aminotransferase (ALT or SGPT), and aspartate aminotransferase (AST or SGOT) to assess any signs of toxicity. According to the applied manufacturer’s instructions, all biochemical markers were evaluated using identified kits: urea (BioSystems S.A. Costa Brava 30, Barcelona, Spain), creatinine (Biolabo, Cat#80107, France), ALAT (BioSystems S.A. Costa Brava 30, Barcelona, Spain), ASAT (BioSystems S.A. Costa Brava 30, Barcelona, Spain).

### Statistical analysis

Data was presented in triplicate as mean ± SD. One-way analysis of variance (ANOVA) and Tukey’s post hoc test were used to evaluate the difference between the mean values of the studied treatments. The analysis was done for three measurements using GraphPad Prism (Version 7.04, San Diego, CA, USA). Statistical values of *p* ≤ 0.05 were considered significant.

## Results and discussion

### Preparation and optimization of method of preparation of RSV invasomes

Invasomes were used as lipid carriers for the encapsulation of RSV using the thin film hydration method [26]. They were used to improve the aqueous solubility and stability of RSV as well as improving its penetration through deeper layers of the skin hence improving its efficacy against skin cancer.

Particle size and polydispersity index (PDI) are some of the major characteristics that influence the release profile of a drug, its stability, encapsulation efficiency, and cellular uptake [36]. Furthermore, low PDI measurements below 0.3 show narrow size distributions with uniform monodisperse particles. It has been reported that different factors affect the particle size and PDI of invasomes such as the method of preparation as well as the concentration of penetration enhancers, ethanol, and terpenes Thus, to prepare an invasomal formulation with optimum particle size and entrapment efficiency, established concentration of invasomal components from the literature was used but different methods of particle size reduction such as homogenization, probe sonication, and extrusion were assessed. PC and terpene concentrations were kept to a minimum (1% w/w) as an increase in PC concentration caused an increase in PS due to the formation of thick PC bilayers around vesicles [10, 28]. Regarding the use of ethanol, it has been used in different invasomal formulations in a concentration range from 1 to 30% [7, 37, 38]. In our study, ethanol was kept to a minimum in the hydration medium (5% v/v) as it plays a role in the solubilization of the vesicular membrane thus delivering RSV into deeper regions of the skin with minimal side effects, avoiding fluidity of the lipid bilayer and leakage of entrapped RSV [39].

As represented in Table 2, using high shear homogenization at 5000 rpm for 3 cycles, 1.5 min for each (F1) led to the formation of invasomes with PS of 408 ± 45.2 nm and PDI of 0.8 ± 0.74 and %EE of 77%. The effect of homogenization speed on PS and %EE was clear as increasing the speed of homogenization to 6000 rpm (F2) caused a decrease in PS and PDI to 208.75 ± 74 nm and 0.3 ± 0.025, respectively, with no significant change in %EE. When the homogenization speed was further increased to 7000 rpm (F3), PS increased to 582 ± 591 nm, PDI increased to 0.6 ± 0.26, while %EE decreased to 74%. Further increase in homogenization speed to 10,000 rpm (F4) led to a significant decrease in PS to 210 ± 52.9 nm, but on the other hand, PDI increased to 0.525 ± 0.17 and %EE significantly decreased to 37 ± 1.579%. This could be explained due to the breaking down of the vesicles by high shear stress [40]. Regarding the duration of each homogenization cycle, upon increasing the cycle time 1.5 to 3 min each at 6000 rpm for F5 and at 7000 rpm for F6, PS significantly increased to 1800 ± 282.8 nm and 968 nm, respectively, compared to F2. The formulations F5 and F6 were not subjected to entrapment efficiency measurement because of their high particle size.

**Table 2:** In vitro characterization data of PS, PDI, and %EE of the different formulated RSV invasomes (mean ± SD, *n* = 3)

**Formula**	**Method of PS reduction**	**Speed of homogenization**	**No. of cycles**	**PS (nm)**	**PDI**	**EE (%)**
F1	High shear homogenization	5000 rpm	3 cycles, 1.5 min each	408.2 ± 45.2	0.8 ± 0.7	77.1 ± 6.2
*F2	High shear homogenization	6000 rpm	3 cycles, 1.5 min each	208.7 ± 74.2	0.3 ± 0.03	77.7 ± 4.3
F3	High shear homogenization	7000 rpm	3 cycles, 1.5 min each	582.2 ± 59.1	0.6 ± 0.2	74.3 ± 3.4
F4	High shear homogenization	10,000 rpm	3 cycles, 1.5 min each	210.1 ± 52.9	0.5 ± 0.1	37.25 ± 1.5
F5	High shear homogenization	6000 rpm	3 cycles, 3 min each	1800.2 ± 282.8	0.4 ± 0.1	-
F6	High shear homogenization	7000 rpm	3 cycles, 3 min each	968.2 ± 63.54	0.4 ± 0.21	-
F7	Probe sonication	60% amplitude	-	1081.1 ± 54.7	0.4 ± 0.26	76.9 ± 2.3
F8	Extrusion	-	5 cycles	256.5 ± 78.5	0.4 ± 0.52	52.2 ± 7.1
F9	Extrusion	-	9 cycles	388.2 ± 116.9	0.5 ± 0.5	55.3 ± 5.4
F10	Extrusion	-	11 cycles	494.2 ± 86.2	1. ± 10.4	60.1 ± 4.8

*PS* Particle Size, *PDI* Polydispersity Index, *%EE* Entrapment Efficiency Percentage

*F2 is the selected optimized formula

Probe sonication was another technique used for PS reduction. Keeping the ethanol:PBS (pH 7.3) ratio constant at 0.5:9.5 and subjecting the samples to probe sonication (60% amplitude for 5 min at 60 °C) (F7) significantly increased the PS to 1081 nm with PDI of 0.41 and %EE of 76.977 ± 2%. This could be due to the effect of sonication on the breakdown of phospholipids, causing low drug entrapment efficiency and high-size distribution. Moreover, extrusion was also tested as a PS reduction method. Extrusion through a 0.2 µm polycarbonate filter was applied in several cycles as 5, 9, and 11 cycles in F8, F9, and F10, respectively, with the same ethanol:PBS ratio (0.5:9.5). PS increased to 256 nm for F8, 388.2 ± 116.9 nm for F9 and 494 nm for F10, while %EE reduced to 52% for F8, 55% for F9, and 60% for F10. Extrusion cycles higher than 11 cycles were not included as they had no effect on decreasing particle size (data not shown).

In conclusion, RSV exhibited a high %EE in the optimized invasome formula (F2) (more than 75%) where %EE was enhanced due to the presence of PC, terpenes, and ethanol [11]. The optimized formula had a loading capacity of 2.5%. PC helped in drug fixation in the lipid bilayer of vesicles, thus decreasing drug leakage [41, 42]. Ethanol and terpenes also helped in the fixation of the drug in the lipid layer by breaking the hydrogen bonds between the lipid layers leaving space for drug entrapment [39].

Zeta potential is another parameter tested that refers to the electrical surface charge of particles indicating the magnitude of repulsion between the vesicles. The higher the zeta potential value (≥ 30 mV), the higher the physical and chemical stability of the formulation and the lower the ability of aggregation of particles [11]. Zeta potential was measured for the optimal invasomal formula (F2) whether the unloaded or RSV-loaded optimal invasomal formula. Both RSV-loaded and unloaded formulas illustrated high negative charges of −70.4 ± 10.9 mV and −76.7 ± 11.4 mV, respectively. This high negative charge determines the high electrostatic stabilization of invasomes along with low particle aggregation. The high negative zeta potential values were caused by the presence of PC and ethanol. PC contains a negatively charged phosphatidyl group and a positively charged choline group. The phosphatidyl group is arranged to the outside of vesicles, while, the choline group is arranged to the inside of vesicles causing the net negative charge on the surface [7]. Additionally, ethanol gives more negative charges on the surface of vesicles stimulating charge repulsion and preventing particle aggregation [7]. Therefore, the presence of PC and ethanol increased the negative charges [7]. Furthermore, the concentration of terpenes increased the negative charge slightly as reported by Lakshmi et al. [6].

According to the results obtained, the optimum conditions in the preparation of invasomes were using ethanolic PBS in hydration solution in the ratio 0.5:9.5 using a high shear homogenizer at 6000 rpm at 3 cycles, 1.5 min each and 5 min rest in between for optimal particle size reduction. This resulted in the preparation of high negatively charged invasomes (−70.4 ± 10.9 mV) with optimum PS (208.7 ± 74.2 nm), the most appropriate PDI (0.3 ± 0.03), and the highest %EE (77.7 ± 4.3%). On the other hand, unloaded invasomes prepared under the same optimum conditions had a PS of 230.7 ± 45.6, PDI OF 0.2 ± 0.05, and zeta potential of −69.5 ± 9.61 mV.

### Transmission electron microscopy (TEM)

**Fig. 1:**
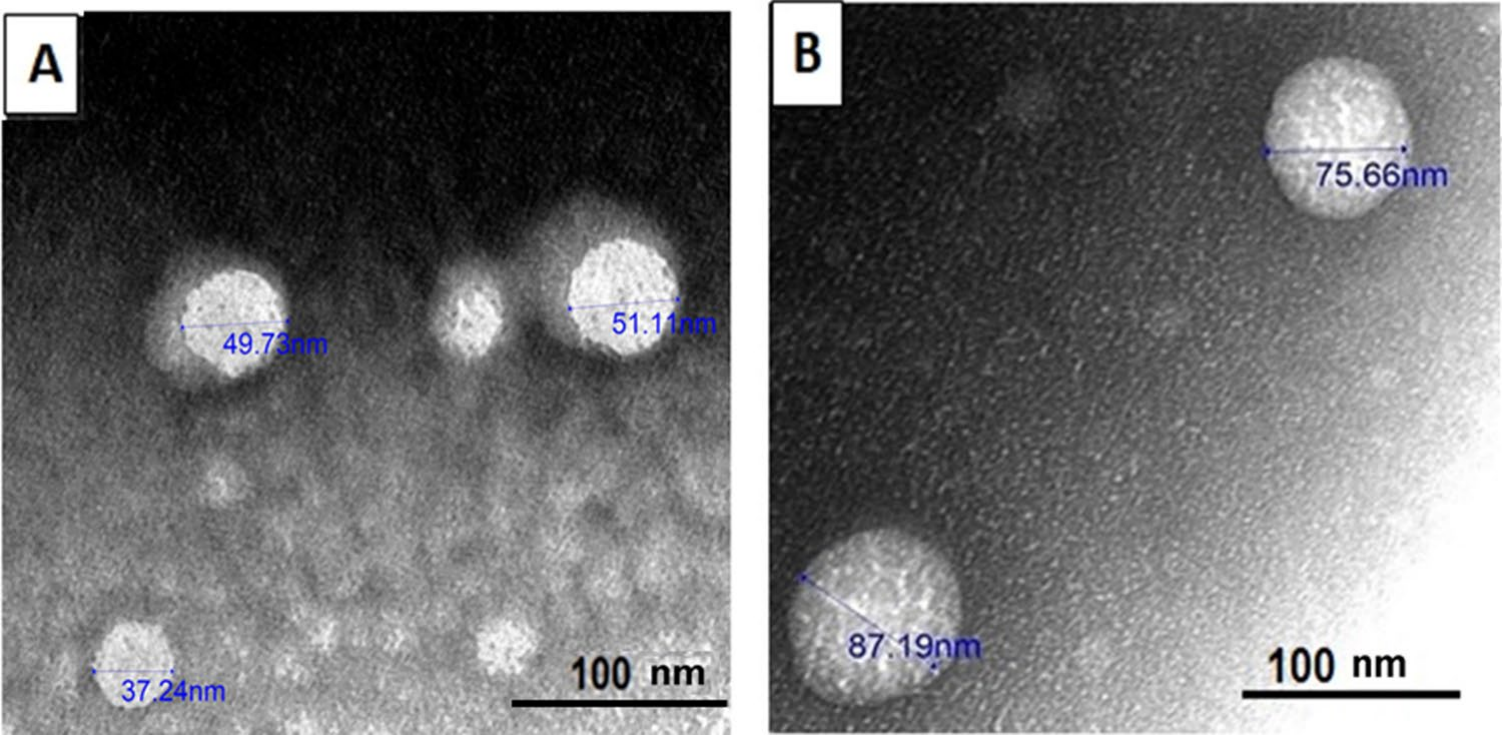
Transmission electron microscope (TEM) images of **A** RSV-loaded invasomes and **B** RSV-unloaded invasomes (placebo) at ×20 K magnification power

The morphology of optimal formula (F2) either for RSV-loaded or unloaded invasomes using TEM showed unaggregated spherical vesicles with PS of 46.1 ± 7.6 nm and 81.4 ± 8.1 nm for RSV-loaded and unloaded invasomes, respectively, as shown in Fig. 1 The PS measured using TEM was relatively lower than those measured by Malvern Zetasizer. This is attributed to the fact that the zetasizer measures hydrodynamic diameter (the nanovesicle surrounded by a liquid layer) while TEM measures the size of air-dried invasomes [27]. The vesicles were unaggregated as a result of the high stability indicated by the high zeta potential with a negative charge [7]. The spherical shape of vesicles could be attributed to the formation of spherical bilayers of lipids upon exposure to an aqueous medium [43].

### Formulation of invasomal-loaded gel

**Fig. 2:**
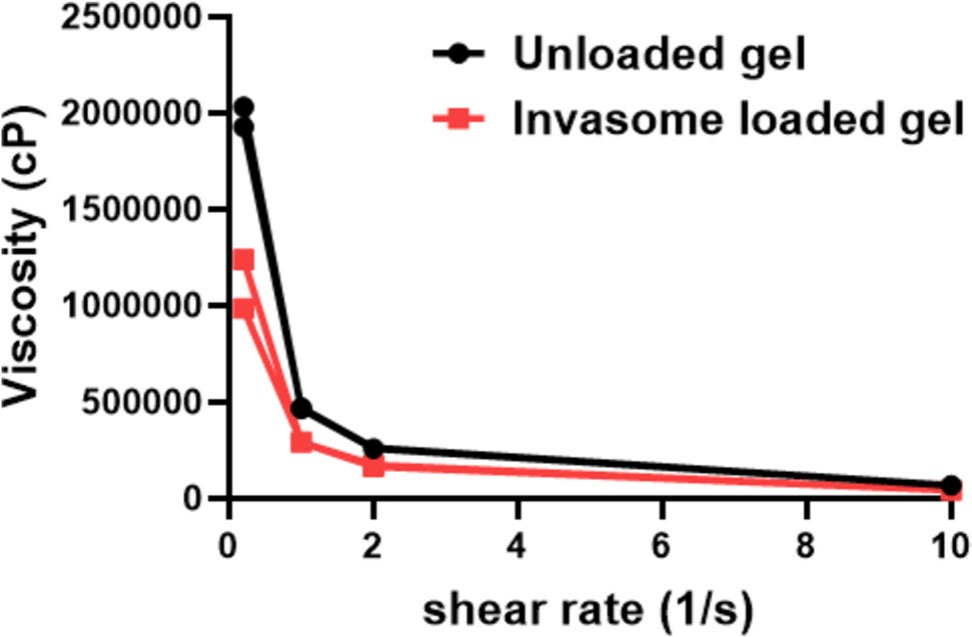
Shear thinning assessment of unloaded and invasomal RSV-loaded gel via viscosity measurement using Brookfield viscometer at 0.1, 0.5, 1, 5, 1, 0.5, 0.1 rpm at room temperature

The gel was successfully prepared using Carbopol 941 as a gelling agent and the drug content was calculated to be 0.5 mg RSV/g gel. Carbopol concentration (3% w/v) was used to adjust the gel viscosity, allow appropriate drug administration and accurate drug dosing, and prolong the time for drug release [44]. The gel also allows the increase in the contact time of formulation on the skin reducing its leakage, as well as improving the stability of invasomes thus its penetration through the skin [45]. Viscosity measurement showed that the gel exhibited thixotropic property where increasing the shear rate caused shear thinning property and pseudoplastic flow for both RSV-invasome loaded gel and unloaded gel as shown in Fig. 2 which is suitable for topical application. Regarding spreadability, the ability of the gel to be spread on the surface of the skin, and a crucial factor ensuring gel consistency, both gels showed optimum spreadability (around 7 cm and 5.8 cm) for unloaded and loaded gel, respectively. These values were in accordance with the spreadability values of Carbopol gels stated in the literature [46] where good spreadability ranges from 5 to 7 cm. The higher the spreadability, the greater the surface area that can be reached by the gel [47]. RSV-loaded invasomal gels showed high spreadability and a decrease in viscosity upon application of certain force, and at the same time had the property of remaining at the application site without drainage.

### In vitro drug release of RSV

**Fig. 3:**
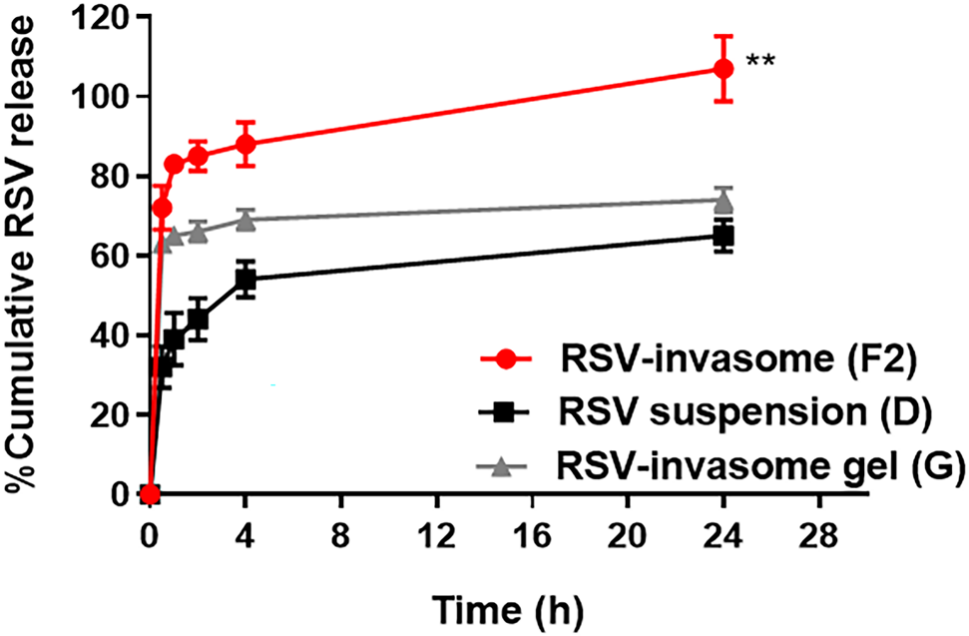
In vitro release profiles of RSV (0.5 mg/mL) in PBS 7.3 at 37 °C and 100 rpm from RSV-invasome dispersion (F2), RSV-invasomal gel (G), and RSV suspension (D). Statistical significance occurred at level of significance where **p ≤ 0.01

As the aim of our work was to develop a formulation with controlled release of RSV, the in vitro release of RSV was assessed. The release of RSV was tested from invasome dispersion (F2) and invasome gel loaded with F2 (G) in comparison to free RSV suspension (D). As shown in Fig. 3, the cumulative in vitro release profile of free RSV suspension exhibited slow release of RSV reaching 32 ± 5.29% in half an hour which increased to reach 44 ± 5.3% after 2 h and 54 ± 4.6% after 4 h. Generally, RSV was released faster when loaded in invasomes where the release of drug from invasomes was influenced by the presence of components as phospholipids, ethanol, and terpenes, as mentioned earlier [7, 11].

RSV showed a slower release from RSV-loaded invasome gel (G) in comparison with (F2) both exhibiting an initial burst effect of RSV followed by controlled release. In the dispersion (F2), the burst effect reached 72 ± 5.5% after 30 min, then increased to 83 ± 2.1% after 1 h which remained increasing at a controlled level till 24 h. However, in RSV-invasomal gel, the initial burst release reached 63 ± 1.5% after 30 min, which was maintained at a controlled pattern for 24 h. The initial burst effect in both the gel and dispersion may be attributed to the presence of free RSV drug fractions on the surface of vesicles which were rapidly released in the first 30 min. This burst drug effect is required to achieve a rapid therapeutic effect by reaching the minimum effective concentration of the drug, followed by a sustained release of deeply encapsulated drug (RSV) inside the vesicular invasomes to prolong the therapeutic efficacy [7]. It has been reported that Carbopol affects the release of drugs via its slow diffusion through the viscous gel matrix decreasing the initial burst release effect of RSV [48].

### Ex vivo skin deposition study

Regarding drug permeation, the percentage of drug deposited in the skin was calculated using HPLC comparing RSV-invasomal gel (G) with RSV-invasome dispersion (F2) and free RSV suspension (D) as shown in Table 3. There was no significant difference between the percentage of RSV deposited in the skin from invasomal gel (G) and RSV-invasome dispersion (60%). On the other hand, only 27% of RSV is deposited in the skin after the application of free RSV suspension.

**Table 3:** Skin permeation and deposition study showing % RSV remaining in the donor compartment, deposited in the skin and permeated through rat skin from RSV-invasome dispersion (F2), RSV-invasomal gel (G), and RSV suspension (D) over a period of 6 h. Statistical significance occurred at level of significance at *p* < 0.05 with mean values a < b

	% donor compartment	% deposited in the skin	% permeated through the skin
RSV dispersion (D)	74.1 ± 1.5^b^	25.9 ± 2.5^a^	0
RSV-invasomal dispersion (F2)	36.5 ± 2.3^a^	60.4 ± 5.4^b^	3.1 ± 0.2
RSV-invasomal gel (G)	33.2 ± 0.5^a^	65.1 ± 5.4^b^	1.7 ± 0.5

High skin deposition of RSV-loaded invasome (either from gel or dispersion) could be attributed to the low PS of invasomes, its high surface area [49], and the presence of penetration enhancers (PC, ethanol and thymol terpene) which act synergistically together as discussed earlier. Terpenes are natural non-irritant volatile oils and are generally recognized as safe (GRAS) [50]. They act as penetration enhancers in low amounts (1%) forming hydrogen bonds with polar head groups within SC disrupting the arrangement of its lipid structure [10]. Thymol was used as a terpene in this study as it is a lipophilic terpene with log P 3.3 [51] and has an antiinflammatory effect [52]. Ethanol decreased the SC rigidity by interacting with the phospholipid polar head group [6] thus making the lipid bilayer of invasomes more flexible to easily penetrate through the intercellular lipids of SC. Thus, the combination of these permeation enhancers improved skin penetration of RSV and enhanced its partitioning through the skin.

### Ex vivo cell culture

#### Cytotoxicity

The cytotoxicity of RSV-loaded invasome liquid dispersion, unloaded invasome liquid dispersion, and RSV solution was assessed to evaluate their anticancer efficacy on squamous skin cancerous cells (SCCs) using MTT assay. A graph of % cell viability was plotted against log concentration of RSV in the 3 different prepared formulations where the IC_50_ of RSV (concentration of drug needed to inhibit cells’ biological activity by 50%) in the 3 different formulations was calculated using GraphPad Prism (version 8.02) [20].

**Fig. 4:**
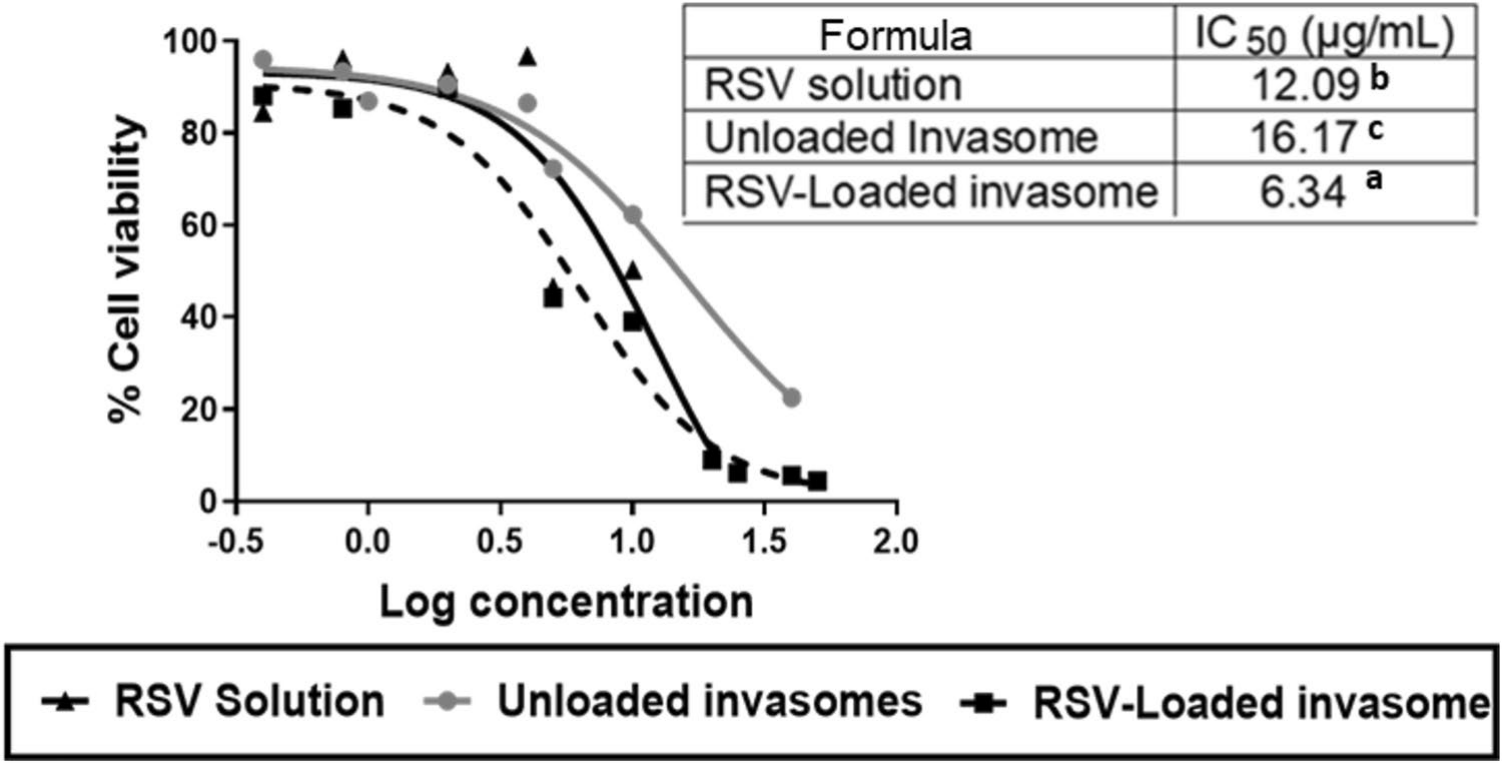
Ex vivo cytotoxicity test of different formulations (RSV solution, RSV-unloaded invasome, and RSV-loaded invasome) on squamous cancerous cells (SCCs) using MTT assay. % Cell viability and IC_50_ (μg/ml) were evaluated in triplicate. The significance level of *p* ≤ 0.05 represents statistically significant differences comparing different groups with mean values a < b < c

As shown in Fig. 4, the RSV solution, unloaded invasome, and RSV-loaded invasome showed an IC_50_ of 12.1, 16.2, and 6.3 μg/mL, respectively. RSV-loaded invasome achieved the highest cytotoxic effect on SCCs exhibited by its lowest IC_50_ value (6.3 μg/mL). This could be attributed to the better solubilization of RSV encapsulated in invasomes [43] and better permeation of invasome vesicles [11]. On the other hand, unloaded invasome and RSV solution resulted in lower cytotoxicity on SCCs with higher IC_50_ of 16.2 and 12.1 μg/mL, respectively, when compared to the RSV-loaded invasomes. This could be due to the inability of RSV solution to permeate via SCCs to achieve its cytotoxic effect. On the other hand, unloaded invasome were capable of penetrating SCCs but without any toxic effect due to the safety of the empty vesicles [52] in the absence of RSV. However, at higher concentrations, unloaded invasomes exhibited slight cytotoxicity due to apoptosis-inducing properties of phospholipid used via reactive oxygen species (ROS) generation as well as the direct disturbing effect of PC on the cell membrane leading to its fluidity and leakage [20].

#### Cellular uptake

**Fig. 5:**
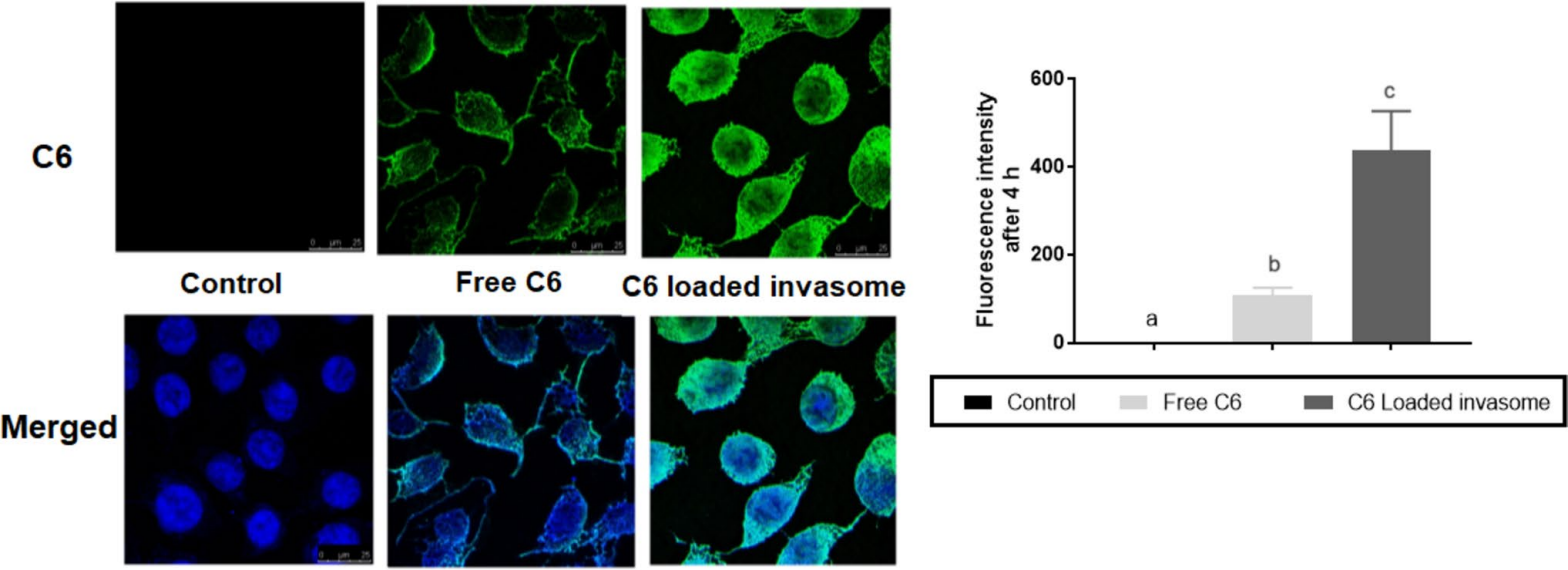
Fluorescence confocal laser scanning microscope (CLSM) photographs and graph illustrating the cellular uptake of coumarin-6 (C6)-loaded invasomes and free C6 solution in comparison with the control after 4 h of incubation. The green color of fluorescence represents C6 dye while the blue color of fluorescence represents the C6 merged with the nucleus which was stained by Hoechst 33,342. The significance level of *p* ≤ 0.05 represents statistically significant differences comparing C6-loaded invasome and free C6 solution with the control with mean values a < b < c

The cellular uptake study was done using the green lipophilic fluorescent dye; coumarin-6 (C6) as a model drug [27] to evaluate the cellular uptake of C6-loaded invasomes and free C6 solution in SCCs using a confocal laser scanning microscope (CLSM), as illustrated in Fig. 5. After incubation of 4 h, the fluorescent intensity of C6-loaded invasomes was significantly higher than that of the C6 solution and the control (*p* ≤ 0.0001). Owing to the highly entrapped C6 inside the invasomes which highly permeated into SCCs, revealing higher cellular uptake of C6-loaded invasomes thus improved skin penetration and internalization in squamous carcinoma cells compared to free C6 solution. Improvement of cellular uptake of invasomes was due to its low particle size and high surface area, the presence of phospholipid bilayer which had a similar structure as that of the cell membrane [53], along the penetration enhancers (ethanol and terpenes) which caused high flexibility and deformability of the invasome lipid bilayer [7].

### In vivo characterization of the anticancer effect of RSV-loaded invasomal gel

Based on the previous results, in vivo topical application of RSV-loaded invasomal gel on Erlich-induced skin cancer mice models was performed to evaluate the antitumor effect and cytotoxicity of RSV encapsulated in invasome delivery system on skin tumors. Groups evaluated were group 1 (Gp1) for healthy negative control mice, group 2 (Gp2) for positive control (untreated cancerous) mice, group 3 (Gp3) for blank gel-treated mice, group 4 (Gp4) for unloaded invasomal gel-treated mice, and group 5 (Gp5) for drug-loaded invasomal gel-treated mice. RSV suspension in gel was not assessed as a treatment group based on its poor deposition in the skin shown in the “Results and discussion” and “Ex vivo skin deposition study” sections.

#### Skin tumor volume measurement

**Fig. 6:**
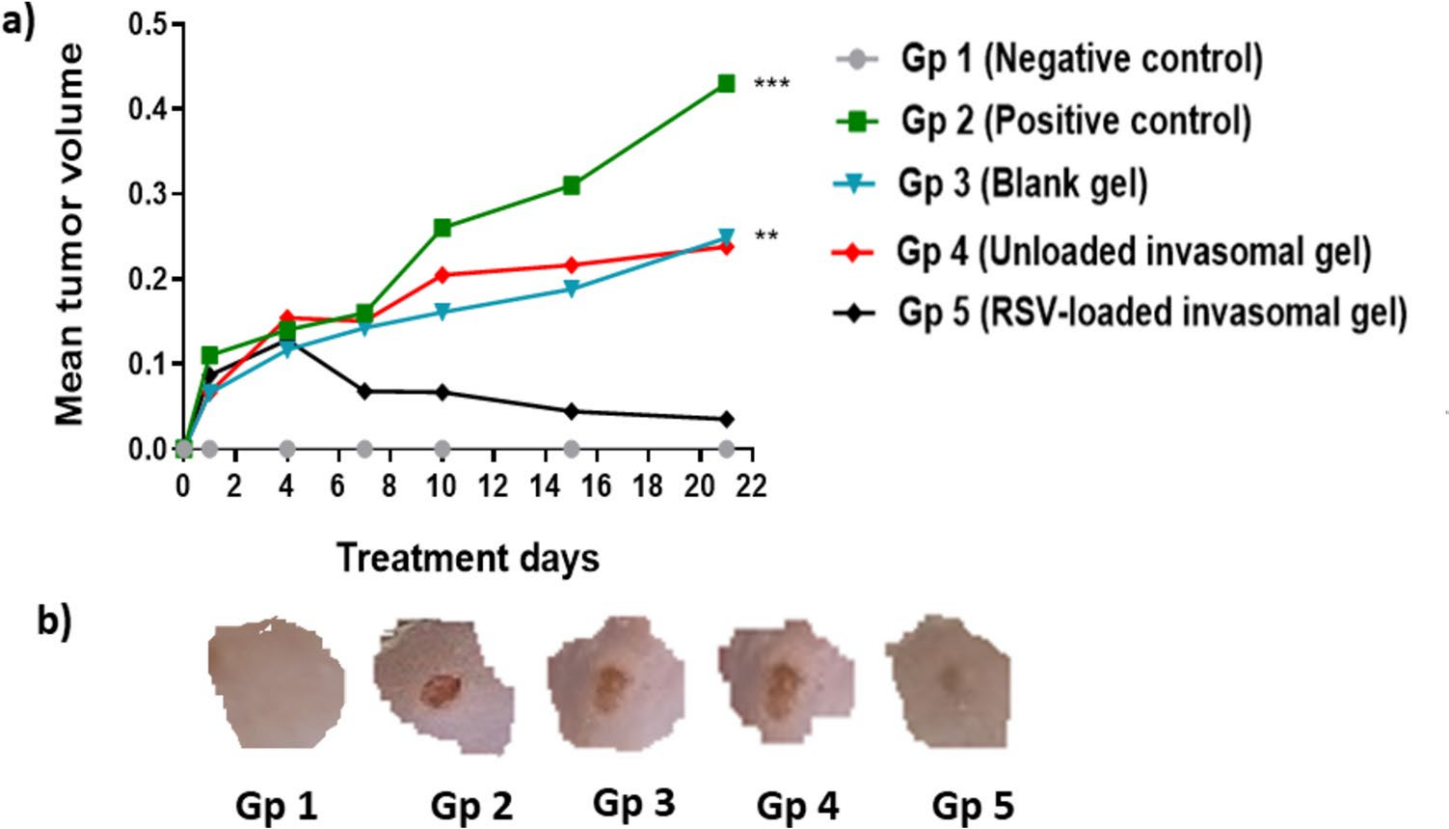
**a** Skin tumor volume (mean) at different treatment days (treatment days 1, 4, 7, 10, 15, 21). Gp 1 is the healthy group, gp 2 is the untreated cancerous group, gp 3 is treated with blank gel, gp 4 is treated with RSV-unloaded invasomal gel, and gp 5 is treated with RSV-loaded invasomal gel. **b** Representative photographs of mice showing tumor volumes in different treatment groups (**1**–**5**) 21 days post-treatment. Each image represents one mouse in each group**.** Statistical significance occurred at level of significance where ** *p* ≤ 0.01 and ***p ≤ 0.001

Measurement of tumor volume has been used to predict cancer progression [54]. As shown in Fig. 6, the tumor was measured during the treatment period on days 1, 4, 7, 10, 15, and 21 (termination day). Skin tumor volumes increased after tumor induction in all groups in comparison to gp 1 (healthy mice). The untreated group (gp 2) showed the highest tumor size compared to all other treated groups. A remarkable reduction in mean tumor size was detected in gp 5 (treated with RSV-loaded invasomal gel) as it showed the smallest tumor volume with a ninefold reduction compared to gp 2 (untreated) 21 days post-treatment. The rate of tumor progression was much slower in gp 3 (blank gel) and 4 (unloaded invasomal gel) compared to gp 2 (untreated group) but higher than that of gp 5 (treated with RSV-loaded invasomal gel). These results proved the high antitumor efficacy and tumor growth inhibitory effect elicited by groups treated with RSV-loaded invasomal gel.

#### Quantitative analysis of cancer biomarkers on gene and protein level

**Fig. 7:**
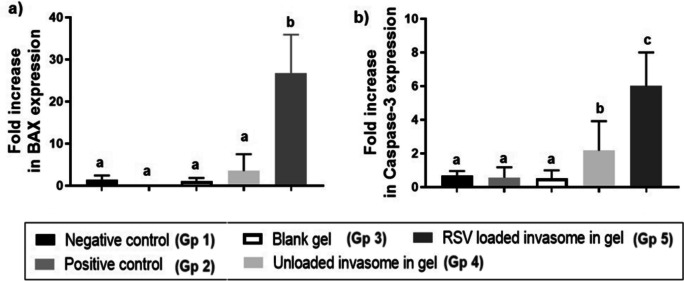
**a** Fold increase in BAX gene expression. **b** Fold increase in Caspase-3 gene expression, determined in all mice groups (negative control, positive control, blank gel, unloaded invasomal gel, and RSV-loaded invasomal gel) using RT-PCR analysis. The significance level of *p* ≤ 0.05 represents statistically significant differences between different treatment groups with mean values a < b < c

RT-PCR analysis of BAX and Caspase-3 gene levels was performed to measure the apoptotic ability of RSV in the different treatment groups thus further proving the cytotoxic effect of RSV as an anticancer drug on the gene level. Both BAX and caspase levels were used as biomarkers for apoptosis as BAX regulates the apoptosis process as a proapoptotic protein for tumor suppression [55, 56]. Caspase-3 is also an important gene for apoptosis induction [57], thus, analyzed to prove the anticancer efficacy and potency of RSV drug on mRNA levels [27]. Therefore, measuring the fold increase of the Caspase-3 and BAX gene levels could be informative about the potency of the formula used. As shown in Fig. 7, mice treated with RSV-loaded invasomal gel (Gp 5) exhibited a 26-fold increase in BAX gene level and a sixfold increase in Caspase-3 gene level than the unloaded invasomal gel-treated group (Gp 4). This proves the improvement in the anticancer efficacy of RSV upon encapsulation in invasomes. The blank gel-treated group (Gp 3) showed no significant change in BAX and Caspase-3 levels, in comparison to negative and positive control groups (Gp 1 and 2) indicating no effect on the apoptosis pathway.

**Fig. 8:**
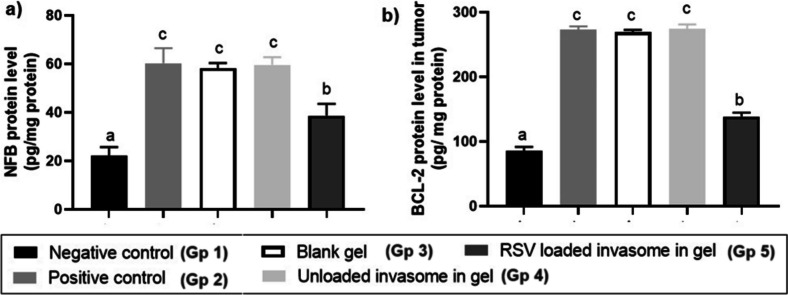
Quantification of NFKB and BCL2 protein levels in different treated groups, where each bar represents mean ± SD of both proteins of 8 mice in each group. Group 1 is the healthy group, group 2 is the untreated cancerous group, group 3 is the group treated with blank gel, group 4 is the group treated with RSV-unloaded invasomal gel, and group 5 is the group treated with RSV-loaded invasomal gel. The significance level of *p* ≤ 0.05 represents statistically significant differences between different treatment groups using means where a < b < c

As an anticancer drug, RSV is also known to suppress inflammatory markers such as TNF-alpha, Cox-2, IL-1, IL-6, Bcl2, and nuclear factor-kappa B (NF-kB) levels [58]. In the current study, the level of NF-kB and Bcl2 protein was evaluated. As shown in Fig. 8, on the protein level, positive (untreated cancerous group) elicited the highest NF-kB and Bcl2 protein levels (60.3 ± 6.22 pg/mg and 273.33 ± 4.73 pg/mg, respectively). Significant reduction in both NF-kB and Bcl2 levels was observed in group 5 (RSV-loaded invasomal gel-treated) to reach 38.6 ± 4.93 pg/mg and 138.67 ± 6.03 pg/mg for NF-kB and Bcl2, respectively. Accordingly, RSV-loaded invasomal gel succeeded in stimulating its antitumor effect topically via reduction of NF-kB and Bcl2 protein levels.

#### Histopathological examination

**Fig. 9:**
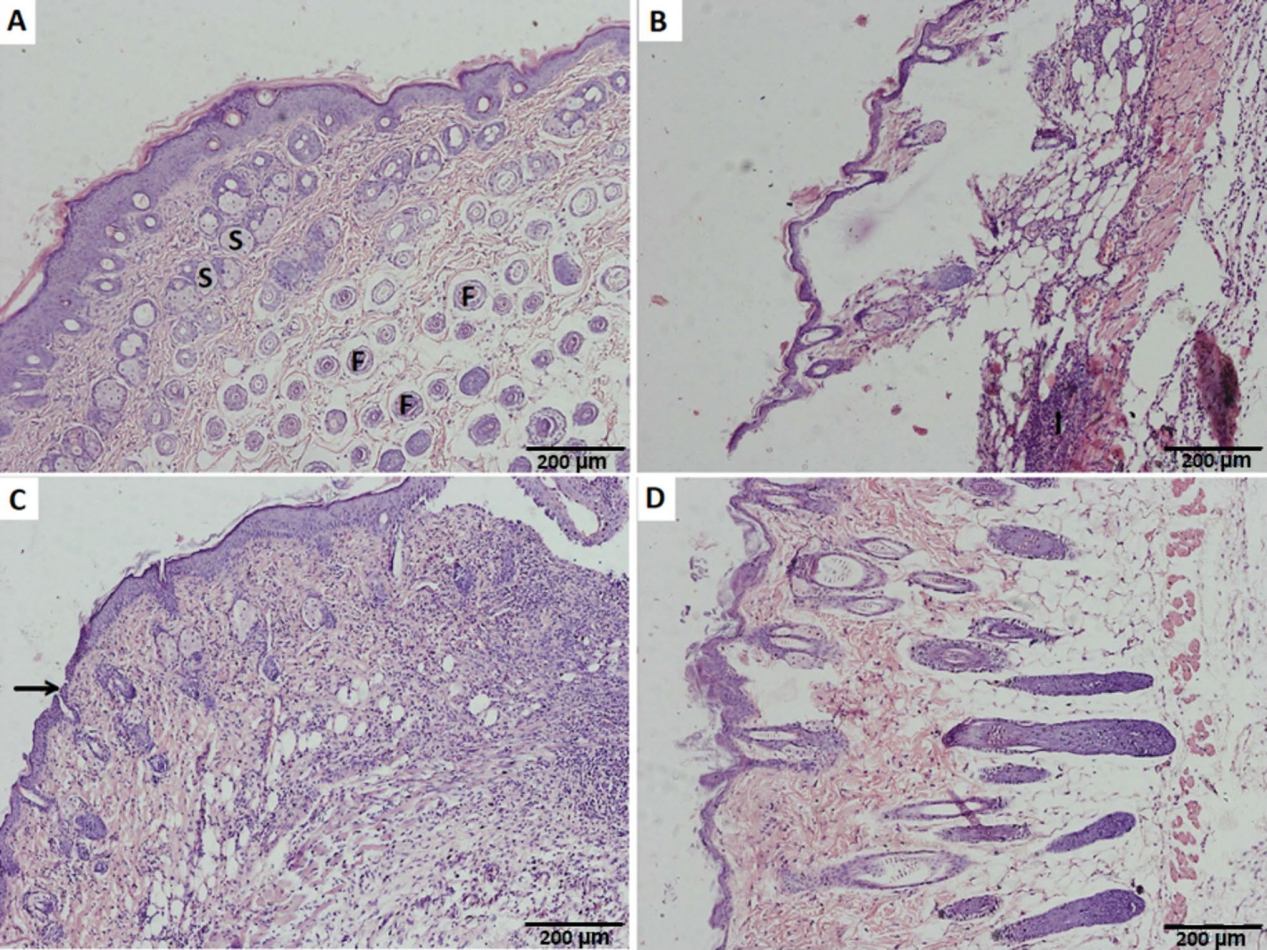
Photomicrographs of mice’s skin sections for **A** G1, negative control; **B** G2, untreated cancerous positive control; **C** G4, RSV-unloaded invasomal gels treated groups; and **D** G5, treated with RSV-loaded invasomal gel). All images were shown at magnification ×200

Histological examination of skin tumor by staining with hematoxylin and eosin (H&E) stain was used to assess the antitumor effect of treated formulations in comparison with the untreated group. As shown in Fig. 9, healthy groups (A) showed clear epidermis and dermis with hair follicles (f) and associated sebaceous glands (s). On the other hand, positive untreated cancerous groups (B) revealed destruction of the normal skin architecture with cellular infiltration (I). Upon treatment of mice with RSV-loaded invasomal gel (D), restoration of normal skin structure occurred indicating its clinical safety, deep permeation ability, and its efficiency in the treatment of skin tumor. Nevertheless, groups treated with unloaded invasomal gels (C) showed no improvement in skin tumor as the epidermis appeared with no keratinization (arrow), and the dermal tissue was thickened with sparse destructed hair follicles and sebaceous glands. Groups treated with blank gels(G3) showed similar results to that of unloaded invasomal gels (C) so no separate representative image of such group was added.

#### Toxicity assessment using blood biochemical assay

Urea and creatinine are considered one of the markers to test for renal toxicity as they are indicators of renal function. Urea elimination by the kidney represents the major route for nitrogen excretion. Interconversion of phosphocreatine and creatine is a particular feature of the metabolism processes of muscle contraction. Creatine and phosphocreatine partially convert to a waste product, creatinine. Thus, the amount of creatinine produced is related to muscle mass and body weight. Elevation of urea and creatinine indicates improper renal function and renal toxicity. On the other hand, ALT and AST are considered the main hepatic markers to test for liver toxicity. Elevation in ALT and AST indicates developing tumors in mice affecting many organs as the liver [59].

**Fig. 10:**
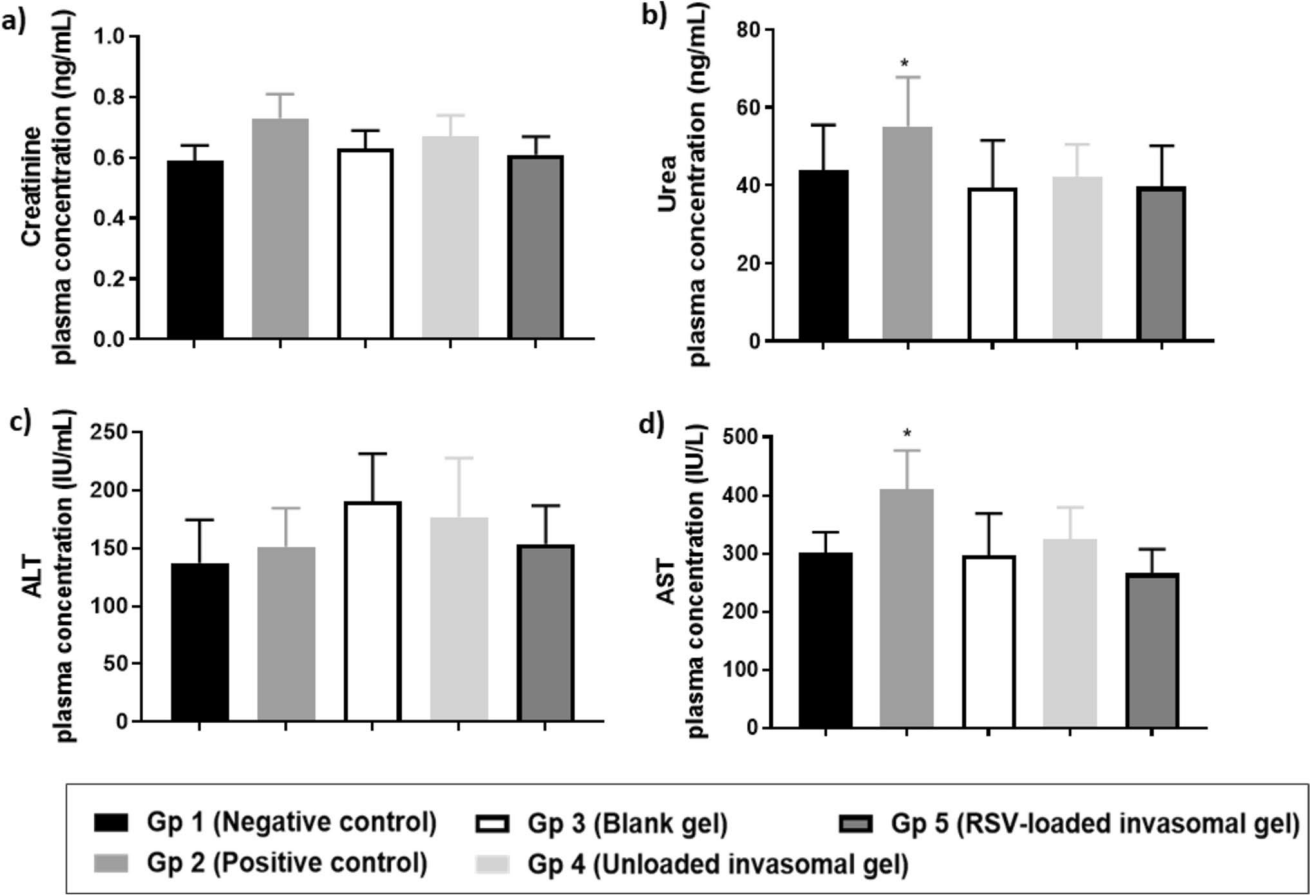
Different plasma biochemical markers such as **a** creatinine, **b** urea, **c** ALT, and **d** AST of all experimental groups after termination. Each bar represents mean ± SD (*n* = 8). Group 1 is the healthy group, group 2 is the untreated cancerous group, group 3 is the group treated with blank gel, group 4 is the group treated with RSV-unloaded invasomal gel, and group 5 is the group treated with RSV-loaded invasomal gel

As illustrated in Fig. 10, gp 2 (untreated cancerous) showed the highest toxicity compared to other groups where levels of urea of 55.2 ± 12.6 ng/mL, creatinine of 0.7 ± 0.1 ng/mL, ALT of 151.5 ± 33.1 IU/mL, and AST of 410.5 ± 66.3 IU/mL were determined. On the other hand, gp 5 presented the lowest toxicity, where it showed urea level of 40.8 ± 15.89 ng/mL, creatinine level of 0.7 ± 0.1 ng/mL, ALT level of 153.8 ± 33.2 IU/mL, AST level of 289 ± 59.4 IU/mL. According to our blood biochemical assay results, treatment with RSV-loaded invasomal gel did not cause liver toxicity or renal toxicity.

## Conclusion

This study highlights the pronounced effect of topical application of RSV when loaded in an invasome formulation to overcome the SC barrier and the limitations of RSV and allow local anticancer treatment. Optimized RSV-loaded invasome possessed low particle size, PDI, high entrapment efficiency, and negatively charged zeta potential influenced by the presence of penetration enhancers (PC, ethanol, and thymol) to allow high skin penetration and anticancer effect of RSV for topical skin cancer treatment. Loading the optimized invasomal formula in a gel helped in controlling the release of RSV release over 24 h. It also exhibited the highest skin deposition percentage (65%) due to the activity of penetration enhancers and low PS of invasomes. Moreover, in vivo Ehrlich tumor-bearing mice models treated with topical RSV-loaded invasome gel exhibited a high antitumor effect against skin carcinogenesis with no liver or renal toxicity via performed blood tests. BAX and Caspase-3 gene expressions showed 26- and six-fold increase respectively in the group treated with RSV-loaded invasomal gel compared to all other groups indicating improved anticancer efficacy of RSV.

In conclusion, invasomes are promising novel safe lipid-based nanosystems for topical RSV delivery having high skin penetration ability and anticancer effect in the treatment of skin cancer with minimal side effects. Yet, further exploratory studies are recommended to elaborate on the anticancer effect of the established formulation with the ultimate goal of implementing such laboratory-based treatment as a bedside regimen. Preclinical studies on larger groups of animal types and humans should be performed to strengthen the research journey from the bench to the bedside.

## Data Availability

The authors confirm that the data for this study’s findings are available within the article.

## Declarations

### Animal studies

Animal studies were performed following the regulations of the National Research Council’s Guide for the Care and Use of Laboratory Animals and approved by the Ethics Commission of Medical Research, Faculty of Pharmacy, Alexandria University Institute (ALEXU-IACUC AU 062021971108). Animals’ distress was reduced following the internationally accepted principles for laboratory use and care of the International Council of Laboratory Animal Science (ICLAS).
